# Pyoderma gangrenosum and cobalamin deficiency in systemic lupus erythematosus: a rare but non fortuitous association

**DOI:** 10.1186/s41927-021-00177-4

**Published:** 2021-03-03

**Authors:** Sing Chiek Teoh, Chun Yang Sim, Seow Lin Chuah, Victoria Kok, Cheng Lay Teh

**Affiliations:** 1 Department of Medicine, Sarawak General Hospital, Kuching, Sarawak Malaysia; 2 Faculty of Medicine and Health Sciences, University Malaysia Sarawak, Jalan Datuk Mohammad Musa, 94300 Kota Samarahan, Sarawak Malaysia; 3 Department of Medicine, Rheumatology Unit, Sarawak General Hospital, Kuching, Sarawak Malaysia

**Keywords:** Systemic lupus erythematosus (SLE), Pyoderma gangrenosum (PG), Cobalamin deficiency, Anaemia

## Abstract

**Background:**

Pyoderma gangrenosum (PG) is an uncommon, idiopathic, ulcerative neutrophilic dermatosis. In many cases, PG is associated with a wide variety of different disorders but SLE in association with PG is relatively uncommon. In this article we present the case of a middle aged patient with PG as the initial clinical presentation of SLE. We also provide a brief review of cobalamin deficiency which occurred in our patient and evidence-based management options.

**Case presentation:**

A 35 years old man presented with a 5 month history of debilitating painful lower limb and scrotal ulcers. This was associated with polyarthralgia and morning stiffness involving both hands. He also complained of swallowing difficulties. He had unintentional weight loss of 10 kg and fatigue. Physical examination revealed alopecia, multiple cervical lymphadenopathies, bilateral parotid gland enlargement and atrophic glossitis. There was Raynaud’s phenomenon noted over both hands and generalised hyper-pigmented fragile skin. Laboratory results disclosed anaemia, leukopenia, hyponatraemia and hypocortisolism. Detailed anaemic workup revealed low serum ferritin and cobalamin level. The autoimmune screen showed positive ANA, anti SmD1, anti SS-A/Ro 52, anti SSA/Ro 60, anti U1-snRNP with low complement levels. Upper gastrointestinal endoscopy with biopsies confirmed atrophic gastritis and duodenitis. Intrinsic factor antibodies and anti-tissue transglutaminase IgA were all negative. Punch biopsies of the leg ulcer showed neutrophilic dermatosis consistent with pyoderma gangrenosum. Based on the clinical findings and positive immunologic studies, he was diagnosed as systemic lupus erythematosus. His general condition improved substantially with commencement of corticosteroids, immunosuppressants and vitamin supplements.

**Conclusions:**

We report a case of PG as the first manifestation of SLE which was treated successfully with immunosuppressants and vitamin supplements. Our report highlighted the need to consider connective tissue diseases such as SLE in a patient presenting with PG in order for appropriate treatment to be instituted thereby achieving a good outcome.

## Background

Pyoderma gangrenosum (PG) is a rare inflammatory and ulcerative disorder of the skin which is often associated with an underlying systemic disease in 50 to 70% of cases [1]. The underlying disease primarily include inflammatory bowel diseases, arthritides, IgA monoclonal gammopathies, and myeloid haematological malignancies. However, pyoderma gangrenosum may also occur on its own [1]. SLE and pyoderma gangrenosum is an uncommon association [1, 2]. We found at least 25 cases of pyoderma gangrenosum associated with SLE in the literature [3]. The diagnosis of PG is made by excluding other causes of similarly appearing cutaneous ulcerations including infections, malignancies, vasculitides, vasculopathies, venous insufficiencies and trauma [1].

Anaemia is a common presentation in rheumatological diseases [4]. The prevalence of anaemia for patients with SLE was reported as 18–80% [5]. Anaemia of chronic disease (ACD) is the most common type [6, 7]. Low cobalamin level was reported in certain SLE patients [8], however, pernicious anaemia is a rare association with SLE [9, 10].

We herein report an atypical case of SLE who presented with PG and cobalamin deficiency. We also discussed the possible pathophysiologic links and performed a literature review of previously published cases. The particularity of this case resides in its presentation and the dramatic improvement after treatment of the underlying aetiology.

## Case presentation

A 35 years old man was hospitalised for multiple, painful and debilitating ulcers over both lower limb and scrotum. It initially started as a small wound over his right shin which gradually grew in size. Subsequently multiple similar lesions appeared extensively on both lower limb and scrotum over a period of 5 months. He was treated with multiple courses of antibiotics to no avail.

Additionally, he had 6 months history of polyarthralgia involving the inter-phalangeal joints of both hands, knees and elbows. He reported early morning stiffness of both hands with discolouration and numbness of his fingers upon exposure to cold temperatures. He also complained of bilateral lower limb numbness. He had significant impairment to his activities of daily living as a consequence of these symptoms. Besides that, he also complained of dysphagia, unintentional weight loss of 10 kg and fatigue.

There was no fever, recurrent oral ulcers, photosensitivity rash and history of gritty or red eyes. He denied chronic cough or night sweats. He had no abdominal pain or alteration in bowel habits. Review of other systems were unremarkable. Family and past medical history were insignificant. He denied alcohol intake, smoking, or use of recreational drugs. He was not a vegetarian and consumed meat regularly. There was no known food intolerance, medication allergies and surgical history.

**Fig. 1:**
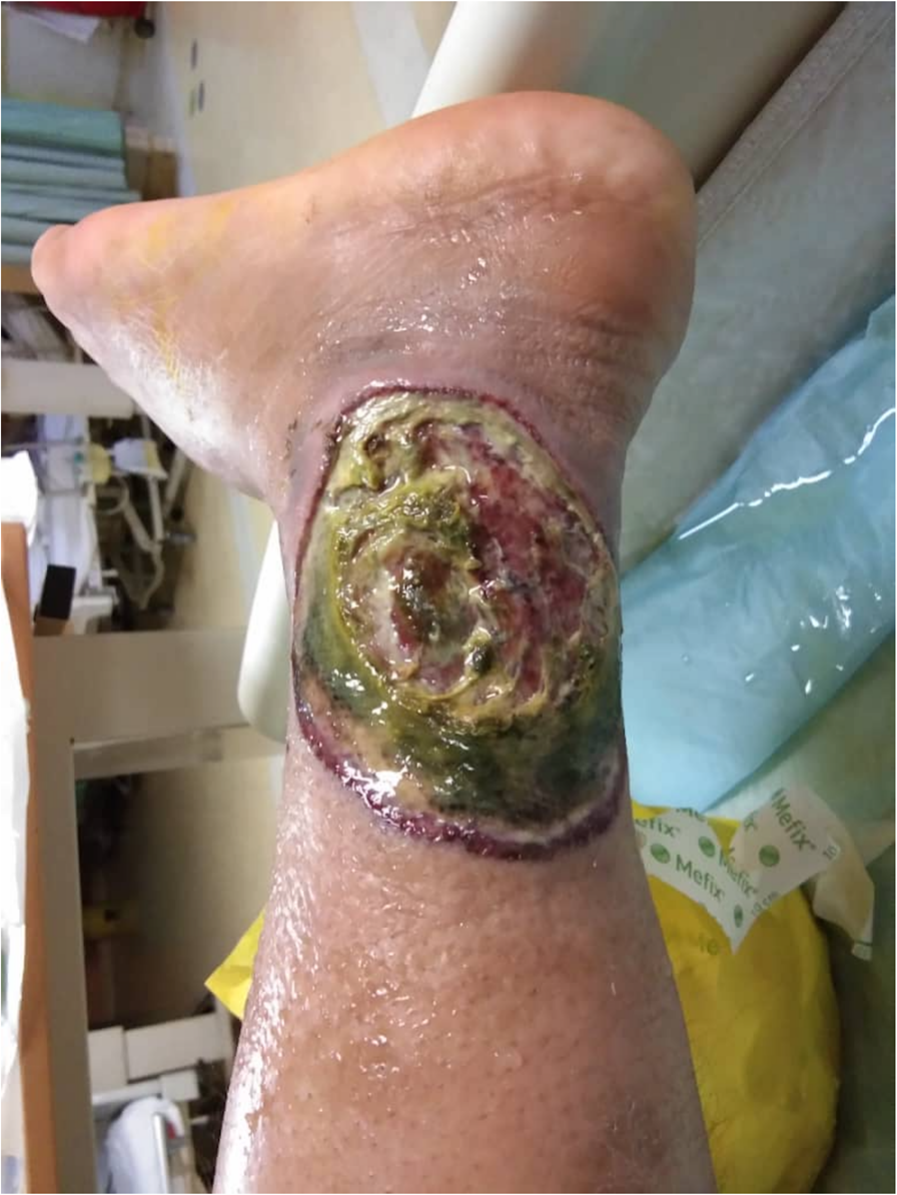
Lesion over posterior aspect of right lower limb with necrotic base and red-blue border

On examination, the patient was afebrile and normotensive with a pulse rate of 110 bpm. There was clear evidence of alopecia, generalised hyper-pigmented fragile skin, depapillation of tongue and bilateral parotid swelling. Raynaud’s phenomenon was noted over both hands. Multiple tiny lymph nodes were palpable over bilateral cervical and supraclavicular areas which were soft and mobile. There were multiple ulcers on his scrotum and both legs of varying ages indicating chronicity with the largest measuring 10 × 5 cm. The ulcers were irregular in shape, had erythematous-purplish borders with a yellowish necrotic base. However, the legs were warm and well perfused, with distinctly palpable peripheral pulses. There was no nodular lesions or thrombophlebitis over the lower extremities. Neurological examination of his lower limbs revealed symmetrical paraesthesia distally with loss of vibratory sensation and diminished proprioception. Respiratory, cardiovascular and abdominal examinations were normal (Fig. 1).

Laboratory investigations revealed haemoglobin of 8 g/dl, mean corpuscular volume (MCV) of 84 fL, white blood cells of 6.27 × 10^3/μL with a lymphocyte count of 1.02 × 10^3 /μL. A peripheral blood film showed normochromic, normocytic anaemia with no evidence of haemolysis or abnormal blast cells. Anaemic work up showed low serum iron of 3.7 μmol/L, total iron binding capacity value of 25.3 μmol/L, low transferrin saturation of 14.6%, serum ferritin of 100 ng/ml, low cobalamin level of 176 pg/mL and normal folate level of 16.3 pg/mL. Serum cortisol level was low at 124.6 nmol/L whilst sodium and potassium levels were also low at 130 mmol/L and 3.0 mmol/L respectively. Liver function test showed low albumin level at 27 g/L with slightly raised AST at 63 U/L. Renal profile and coagulation screening were normal. Antinuclear antibody (ANA) was positive at a titre of 1:5120 which has a speckled pattern. Other autoimmune screening showed positive anti-SSA/Ro 60, anti-SSA/Ro 52, antiSmD1, and anti U1-snRNP. C-ANCA, p-ANCA, double stranded DNA, anti-SSB/La and rheumatoid factor were not detected. Complement 3 and 4 were below the reference range. Both inflammatory markers were elevated with CRP of 219 mg/L and ESR of 154 mm/hr. Serum protein electrophoresis displayed no paraprotein band or immunoparesis. Quantitative serum immunoglobulin test were normal. The serological tests for various infectious agents (HBsAg, anti HCV ab, anti HIV 1 + 2 ab and treponemal tests) were negative. Intrinsic factor antibodies and anti-tissue transglutaminase IgA were below detection limits.

Cultures and sensitivities from the leg ulcers were positive for *Proteus mirabilis*. He was treated with a two-week course of intravenous amoxicillin/clavulanic acid. Histopathological examination of the leg ulcers demonstrated dense inflammatory cells infiltrate with neutrophil predominance. There was no evidence of necrotising vasculitis, vascular thrombosis, squamous dysplasia or invasive malignancy. No atypical mycobacteria or yeast were detected. An oesophagogastroduodenoscopy and colonoscopy was done which revealed atrophic gastritis, duodenitis and normal colonic mucosa. Campylobacter-like organism (CLO) test was negative. Histologically, the biopsies of small bowel showed well-formed and irregularly spaced glands secondary to expansion of lamina propria by lymphoplasmacytic inflammatory cell infiltrates. Colonic biopsies were insignificant. Periodic acid-Schiff (PAS) staining was negative. The findings were inconsistent with inflammatory bowel disease, Whipple’s disease, coeliac disease or pernicious anaemia.

Based on clinical features, laboratory investigations, endoscopic and histological findings, he was diagnosed as SLE with pyoderma gangrenosum and cobalamin deficiency. He was treated with prednisolone, hydroxychloroquine, methotrexate, parenteral cyanocobalamin and iron supplements. Noticeable clinical improvement was observed after commencement of recommended treatment. Upon clinic review 1 month later, he reported weight gain, increased level of well-being, great improvement of skin lesions along with significant control of symptoms.

## Discussion and conclusion

PG is part of the neutrophilic dermatosis spectrum of which immunohistological examination reveals neutrophilic predominant inflammatory reaction without evidence of infection [3]. The pathophysiology of PG is still unclear. An increasing body of evidence supports the role of pro-inflammatory cytokines like interleukin (IL)-1-beta, IL-17 and tumour necrosis factor-alpha in the pathophysiology of neutrophilic dermatosis similar to classic monogenic auto-inflammatory diseases [3]. At present, there are no clear guidelines concerning PG treatment in patients with SLE [3].

The therapeutic strategy for neutrophilic dermatosis consists of modulating activation, maturation or migration of neutrophils with treatments such as colchicine, dapsone, corticosteroids and minocycline [3]. Interestingly, medications such as hydroxychloroquine, methotrexate, mycophenolate mofetil and cyclophosphamide used in modulating connective tissue diseases activity like SLE were reportedly effective on neutrophilic skin lesions as well [3]. This observation suggests that SLE and PG may share some common points in their pathogenesis [3].

From the literature search, there are at least 25 case studies of PG associated with SLE [3]. About 72% (18/25) of these cases were diagnosed with SLE prior to the onset of PG, in comparison to 12% (3/25) who presented with PG prior to diagnosis of SLE [3]. Both diseases appeared simultaneously in 16% of the reported (4/25) cases [3]. Active lupus was seen in 12 patients at the time of PG onset, while 8 patients were asymptomatic of SLE [3]. However, in another 2 patients, the criteria of SLE were inconclusive [3]. In our patient, PG appeared simultaneously with active SLE. The occurrence of PG was correlated with the SLE activity in our patient as observed in the majority of the published cases of such association [3]. Main features of the 25 cases of PG associated with SLE and patients characteristics are tabulated in Table 1.

**Table 1:** Main characteristics of 26 SLE patients with pyoderma gangrenosum

No.	Reference	Gender/ Age	Location of lesion (number)	Timing of PG onset compared to SLE diagnosis	Lupus activity at the time of PG onset
1.	Our patient	M/35	Leg (MP)	Simultaneous	Signs of activity
2.	Lebrun [3]	F/32	Face (3)	1 year later	No activity
3.		F/37	Leg (MP)	10 years later	Signs of activity
4.	Gonzalez-Moreno [11]	M/46	Leg (1)	After	Signs of activity
5.		F/63	Foot (1)	After	No activity
6.		F/42	Leg (1)	After	No activity
7.		F/46	Leg (MP)	After	No activity
8.		M/36	Foot (MP)	After	Signs of activity
9.	Hamzi [12]	F/35	Leg (1)	18 years later	No activity
10.	Canas [13]	NS/48	MD	2 years later	MD
11.		NS/28	MD	4 years later	Signs of activity
12.		NS/50	MD	Simultaneous	MD
13.	Husein-EIAhmed [14]	M/36	Foot (1)	8 years later	No activity
14.	Masatlioglu [15]	F/35	Leg (1)	Simultaneous	Signs of activity
15.		F/47	Thigh (1)	10 months before	NA
16.	Hind [16]	F/14 month	Face, ends (MP)	Simultaneous	Signs of activity
17.	Reddy [17]	M/34	Legs (MP)	After	Signs of activity
18.	Waldman [18]	F/35	Leg (MP)	5 years before	NA
19.	Sakamoto [19]	F/55	Trunk/chest/shoulders (MP)	3 years later	Signs of activity
20.	Schmid [20]	F/64	Leg (2)	11 years later	Signs of activity
21.	Holbrook [21]	F/57	Leg (1)	2 years later	No activity
22.	Roger [22]	F/25	Foot (1)	1 month later	Signs of activity
23.	Hostetler [23]	F/27	Trunk,chest, knees (MP)	13 years later	No activity
24.	Pinto [24]	F/35	Leg (1)	15 years later	Signs of activity
25.	Peterson [25]	F/48	Intergluteal and inguinal folds (6)	Simultaneous	Signs of activity
26.	Olson [26]	F/15	Leg (MP)	1 year before	NA

*M* male, *F* female, *MP* multiple, *MD* missing data, *PG* pyoderma gangrenosum, *SLE* systemic lupus erythematosus

The prevalence of anaemia for patients with SLE was reported as 18–80% [5]. Anaemia in these patients consists of anaemia of chronic disease (ACD)(60–80%), iron deficiency anaemia (IDA), autoimmune haemolytic anaemia (AIHA), and anaemia secondary to chronic renal insufficiency [6]. Other less common types of anaemia included pure red cell aplasia (PRCA), pernicious anaemia (PA), and aplastic anaemia [6]. In a study cohort consisting of 132 anaemic patients with SLE, ACD was found in 37.1% of the cases, IDA in 35.%, AIHA in 14.4% and other causes of anaemia in 12.9% of the patients [7]. Based on a study on 42 patients diagnosed with SLE, cobalamin levels were found to be significantly lower in the SLE group compared with a normal control group, eight of whom (18.6%) had serum cobalamin levels equal to or lower than 180 pg/mL [8].

Our patient exhibited a mixed nutritional deficiency anaemia with both cobalamin and iron deficiencies. The possible mechanism for this may be explained by the presence of chronic atrophic gastritis seen endoscopically. Chronic atrophic gastritis results in the destruction of gastric mucosa parietal cells leading to reduced gastric acid secretion [27]. This precipitated the decrement in intrinsic factor production and food iron solubilisation, which eventually caused cobalamin and iron malabsorption [27]. The incidence of atrophic gastritis (AG) in SLE is low [28]. Immunological factors are known to be involved in the aetiology of the disease [28]. However, antiparietal cell antibodies and intrinsic factor antibodies are a rare finding [9]. Intrinsic factor antibodies were present in only 3/30 (10%) SLE patients and 0/45 controls in a study [10]. Pernicious anaemia (PA) is infrequently associated with SLE [9, 10]. To the best of our knowledge, only four cases of PA was recorded among in patients with SLE [10]. This association of uncertain significance between SLE and PA suggested only a minority of cases of cobalamin deficiency in SLE would be related to the anti-intrinsic factor antibody [10]. Future investigations on the mechanisms of the cobalamin deficiency observed in SLE patients are required. This is an important recognition as low serum cobalamin concentrations is a precursor of PA.

The effective treatment for cobalamin deficiency are intramuscular (IM) injections of cyanocobalamin or oral therapy. Approximately 10% of the standard injectable dose of 1 mg is absorbed, which allows for rapid replenishment in patients with severe deficiency or severe neurologic symptoms [29]. High-dose oral replacement (1 mg to 2 mg per day) was as effective as parenteral administration for correcting anaemia and neurologic symptoms [30]. However, there is a lack of data on the long-term benefit of oral therapy when patients do not take daily doses [30]. Therefore, intramuscular cobalamin was recommended for severe deficiency and malabsorption syndromes as in our patient. Oral replacement may be considered for patients with asymptomatic, mild disease with no absorption or compliance concerns [31].

A comprehensive and holistic approach in managing such patients is essential to get a confirmatory diagnosis. A multidisciplinary approach with various subspecialties e.g. rheumatologist, internists, gastroenterologists, dermatologists, etc. is utmost necessary to assist in the diagnosis of this patient. Diagnosis of PG and cobalamin deficiency should raise the possibility of concomitant autoimmune diseases, that could be missed if overlooked, resulting in significant delay in treatment which could be devastating. Overall, our case adds to the pool of knowledge about the pathogenesis, presentation and management of PG and cobalamin deficiency in association with SLE.

## Data Availability

Data are contained within the manuscript.

## Abbreviations

SLE: Systemic Lupus Erythematosus
PG: Pyoderma gangrenosum
ANA: Anti-nuclear antibody
ANCA: Anti-neutrophil cytoplasmic antibody
p-ANCA: Perinuclear anti-neutrophil cytoplasmic antibodies
AST: Aspartate aminotransferase
CRP: C-reactive protein
ESR: Erythrocyte sedimentation rate
